# Vitamin D and Serum Cytokines in a Randomized Clinical Trial

**DOI:** 10.1155/2010/305054

**Published:** 2010-08-12

**Authors:** Eleanor Yusupov, Melissa Li-Ng, Simcha Pollack, James K. Yeh, Mageda Mikhail, John F. Aloia

**Affiliations:** Winthrop University Hospital, Bone Mineral Research Center, 222 Station Plaza North, Suite 350A, Mineola, NY 11501, USA

## Abstract

*Background*. The role of vitamin D in the body's ability to fight influenza and URI's may be dependent on regulation of specific cytokines that participate in the host inflammatory response. The aim of this study was to test the hypothesis that vitamin D can influence intracellular signaling to regulate the production of cytokines. *Subjects and Methods*. This study was a 3-month prospective placebo-controlled trial of vitamin D3 supplementation in ambulatory adults [Li-Ng et al., 2009]. 162 volunteers were randomized to receive either 50 *μ*g/d (2000 IU) of vitamin D3 or matching placebo. 25(OH)D and the levels of 10 different cytokines (IL-2, 4, 5, 6, 8, 10, 13, GM-CSF, IFN-*γ*, TNF-*α*) were measured in the serum of participants at baseline and the final visit. There were 6 drop-outs from the active vitamin D group and 8 from the placebo group. *Results*. In the active vitamin D group, we found a significant median percent decline in levels of GM-CSF (−62.9%, *P* < .0001), IFN-*γ* (−38.9%, *P* < .0001), IL-4 (−50.8%, *P* = .001), IL-8 (−48.4%, *P* < .0001), and IL-10 (−70.4%, *P* < .0001). In the placebo group, there were significant declines for GM-CSF (−53.2%, *P* = .0007) and IFN-*γ* (−34.4%, *P* = .0011). For each cytokine, there was no significant difference in the rate of decline between the two groups. 25(OH)D levels increased in the active vitamin D group from a mean of 64.3 ± 25.4 nmol/L to 88.5 ± 23.2 nmol/L. *Conclusions*. The present study did not show that vitamin D3 supplementation changed circulating cytokine levels among healthy adults.

## 1. Introduction

Vitamin D is produced in the skin when sunlight is absorbed. Thus, vitamin D levels, or serum 25-hydroxyvitamin D (25(OH)D), fluctuate seasonally. 25(OH)D levels are low during the winter in northern latitudes because of decreased amounts of sunlight. A conventional diet usually does not provide adequate amounts of vitamin D. Vitamin D insufficiency results in a number of skeletal and extraskeletal complications. It has been associated with decreased muscle strength [1], breast cancer [2, 3], colon cancer [4], cardiovascular disease [5], and autoimmune disorders such as type 1 diabetes [6], rheumatoid arthritis [7] and systemic lupus erythematosus [7]. Based on vitamin D's role in the basic innate immune defense mechanisms, it has been suggested as an adjuvant treatment of tuberculosis [8]. Levels of 25(OH)D, below 17.8 ng/ml are associated with a 26% increased risk of all-cause mortality in the general population even after adjusting for known CVD risk factors, socioeconomic status, and other characteristics [9]. 

In vitro and in vivo studies show a role for vitamin D as an important component of the innate immune system. The innate immune system provides front-line protection against infectious agents. Expression of vitamin D receptor occurs in different cells of the myeloid and lymphoid lineage. The active form of vitamin D, 1,25-dihydroxyvitamin D (1,25(OH)_2_D), increases the production of endogenous antibiotics called antimicrobial peptides (AMP) in human monocytes, neutrophils, and epithelial cells [10]. AMPs such as defensin and cathelicidin have a broad range of actions against microorganisms, including bacteria, fungi, and viruses. Liu et al. showed that stimulation of toll-like receptors (TLR) 2/1 engages a vitamin D-dependent intracellular circuit that results in the expression of cathelicidin, enhancing the microbicidal capability of the monocyte [8]. Remarkably, the authors also observed that sera from African-American individuals, who are known to have substantially lower serum 25(OH)D levels than whites [11], were inefficient in inducing genetic expression of cathelicidin. When the sera was supplemented with 25(OH)D, cathelicidin levels increased to levels observed in monocytes collected from whites. This suggests that vitamin D insufficiency during the winter may increase susceptibility to infections, particularly viral respiratory infections.

It is estimated that 72% of adults experience at least one URI per year and those adults experience an average of 2.5 URIs per year [12]. Every year 5% to 20% of the U.S. population get the flu [13]. Vitamin D may also play a role in reducing the severity of URI symptoms by regulating specific cytokines that participate in the host inflammatory response. Vitamin D's role in fighting influenza may be dependent on suppression of pro-inflammatory cytokines Interferon gamma, TNF-*α*, and IL-12 in the macrophage [14]. 

1,25(OH)_2_D has been shown to inhibit mononuclear and T lymphocyte cell proliferation by decreasing the production of IL-1*β*, IL-2, IL-6, interferon *γ* (IFN-*γ*), and TNF-*α* [15]. A randomized controlled trial by Schleithoff et al. showed that 50 *μ*g/day (2000 IU/day) of vitamin D3 reduced the inflammatory milieu in CHF patients [16]. 

1,25(OH)_2_D may play a protective role against inflammatory bowel disease as was shown in a mouse model of colitis. Tissue-specific alterations in the expression of cytokines IL-1*β*, IL-10, and IL-17 were demonstrated in Cyp27b1 knockout mice [17]. 

When reviewing the adverse events from our prior study of vitamin D3 supplementation in postmenopausal African American women [18], we noticed a significant difference in the reported incidence of URI symptoms between the active vitamin D group and the placebo group. This finding led us to conclude that higher doses of vitamin D supplementation may protect against viral URI's [19].

Current recommendations for vitamin D intake are based on amounts required to sustain optimal skeletal health. Schleithoff's study and our prior study suggest that optimal function of the innate immune system might require higher doses of vitamin D. In this study, we administered 50 *μ*g/day (2000 IU) of vitamin D3 which is the tolerable upper intake level (UL) for vitamin D for children and adults set by the Food and Nutrition Board of the Institute of Medicine [20]. Experts in the U.S. believe that higher intakes of vitamin D are necessary and that these higher intakes are safe [21, 22].

The role of vitamin D in the body's ability to fight influenza and URI's may be dependent on regulation of specific cytokines that participate in the host inflammatory response. The aim of this study was to test the hypothesis that vitamin D can influence intracellular signaling to regulate the production of cytokines. The levels of 10 different cytokines produced by T cells (Th1, Th2, regulatory T cells, NK cells) and macrophages were measured in the serum of participants at baseline and the final visit.

## 2. Patients and Methods

### 2.1. Subjects

**Figure 1:**
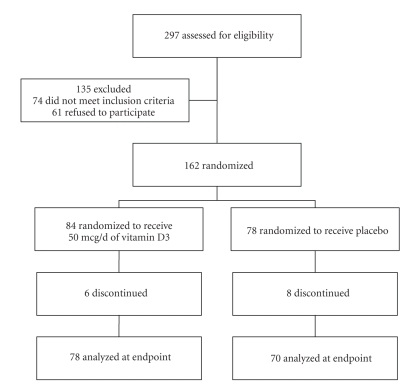
Epidemiology and Infection, Volume 137, Issue 3, 2009 “A randomized controlled trial of vitamin D3 supplementation for the prevention of symptomatic upper respiratory tract infections” by John Aloia. © 2009 Cambridge University Press.

Study participants were recruited from the Long Island, New York community (latitude, 40.7 degrees N) between December 2006 and March 2007 (Figure 1 Flowchart). Volunteers were recruited from local newspaper advertisements, mailing of brochures to community residents, and flyers posted at Winthrop University Hospital medical offices. Patients were eligible for the study if they met the following criteria: ambulatory adult age 18–80 and stable medical condition with no change in medications for 6 months prior to study entry. Exclusion criteria included morbid obesity (body mass index >35 kg/m^2^); current tobacco use; history of hypercalcemia, nephrolithiasis or sarcoidosis; pregnancy; recent hospitalization; current liver or kidney disorders, malignancy and malabsorption; and use of immunosuppressants or medications that interfere with vitamin D metabolism such as phenytoin and carbamazepine. Race determination was by self-declaration. All participants provided written informed consent and the trial was approved by the institutional review board of Winthrop University Hospital. The results of this study have been recently reported [19].

### 2.2. Study Design

This study was a 3-month prospective, randomized, double-blind, placebo-controlled trial of vitamin D3 supplementation in ambulatory adults [19]. Recruitment began from December 2006 to March 2007 and the study was completed in June 2007. The participants were randomly assigned using a computer-generated randomization sequence to receive either 50 *μ*g/d of vitamin D3 or matching placebo. Each subject was sequentially assigned a number upon study entry and the investigators dispensed the corresponding sequentially numbered container of study medication to the subject. All participants and investigators were blinded throughout the study except for the research pharmacist and the statistician. Neither the statistician nor the research pharmacist had any contact with study participants. Eligible subjects underwent a baseline medical history, height and weight measurements, and blood tests. Subjects were seen at 6-weeks and 12-weeks postrandomization. Blood was collected again at the 12-week visit. 25(OH)D and the levels of 10 different cytokines (IL-2, 4, 5, 6, 8, 10, 13, GM-CSF, IFN-*γ*, TNF-*α*) were measured in the serum of participants at baseline and the final visit.

### 2.3. Laboratory Tests

Serum samples were analyzed by Ray Biotech Quantibody Human Th1/Th2 Array 1 kit using glass-chip-based multiplexed sandwich ELISA system. The signals are detected using fluorescence-based detection method. Baseline and study-end serum samples were analyzed in one assay. The well-to-well CV is <20%.

Serum 25(OH)D was measured by a radio-receptor assay from DiaSiorin, Inc (Stillwater, MN). The intraassay variability in our laboratory is 4.1% and interassay variability is 7.0%. Our laboratory participates in the international Vitamin D External Quality Assessment Program (http://www.deqas.org/). Vitamin D3 content was analyzed in an independent laboratory (Vitamin D, Skin, and Bone Research Laboratory, Department of Medicine, Boston University School of Medicine, Boston, MA).

### 2.4. Statistical Analysis

The signed rank test was used to assess the median percent change of each cytokine level within the active vitamin D or placebo group during the 12-week period. The rank-sum test was used to compare median percent change between the active vitamin D and placebo groups. An estimate of the difference of the two medians (active vitamin D-placebo) and the corresponding 95% confidence interval was calculated using the software Confidence Interval Analysis (CIA), Version 2.0.0. We also pooled both groups together and calculated percent changes in 25(OH)D over 12 weeks. Spearman correlations were then calculated between these percent changes in 25(OH)D and percent changes of each cytokine level. Since analysis was done for 10 cytokines, we used the Bonferroni adjustment and results were considered significant at the *P* = .05/10 = .005  level of significance. For all calculations (except for confidence interval analysis), we utilized SAS 9.2 for Windows, SAS Institute Inc., Cary, N.C.

## 3. Results

### 3.1. Baseline Characteristics

The baseline characteristics and laboratory values of the study population are summarized in Table 1 [19]. *T*-tests comparing active to placebo patients at baseline did not reveal any differences between the groups. The baseline 25(OH)D levels ranged from 16 to 156 nmol/L with a mean level of 63.7 ± 28.7 nmol/L in the study population. At baseline, 23% of the active patients exceeded 75 nmol/L.

### 3.2. Adherence

Adherence (defined as the ratio of the number of pills consumed to the number of days in the study) ranged from 59% to 100%. Mean compliance was 94% ± 9%. Adherence did not significantly differ between the active and placebo groups.

### 3.3. Cytokine Levels

There were no statistically significant differences between the two groups with respect to median percent differences for each cytokine. The only exception was for IL-10, where the percent change for the active vitamin D group was −70.4% versus only −49.6% for placebo (*P* = .02). However, the Bonferroni adjustment would need to be removed in order to obtain significance at *P* < .05.

In the active vitamin D group, we found a significant median percent decline in levels of GM-CSF (−62.9%, *P* < .0001), IFN-*γ* (−38.9%, *P* < .0001), IL-4 (−50.8%, *P* = .001), IL-8 (−48.4%, *P* < .0001), and IL-10 (−70.4%, *P* < .0001). In the placebo group, there were significant declines (but slightly smaller compared to the active vitamin D group) for GM-CSF (−53.2%, *P* = .0007) and IFN-*γ* (−34.4%, *P* = .0011). For each cytokine, there was no significant difference in the rate of decline between the two groups. 25(OH)D levels increased in the active vitamin D group from a mean of 64.3 ± 25.4 nmol/L to 88.5 ± 23.2 nmol/L. At the end of the study, 73% of the active vitamin D group patients exceeded 75 nmol/L. Spearman correlation analysis showed no significant correlations for any of the 10 cytokines: the strongest correlations were *r* = −0.096 (*P* = .30) for IL-8 and *r* = 0.079 (*P* = .39) for IL-5.

Cytokine results as described above are summarized in Table 2. We provide in Table 3, for each cytokine, the difference of the median percent change (Active vitamin D-Placebo) and the corresponding 95% confidence interval. We note that for nine of the ten cytokines, the estimated difference of medians between the two groups was within ±10%, which would likely represent a clinically insignificant difference. The only exception was for cytokine IL-10, where the group difference was statistically significant (*P* = .02 before the Bonferroni adjustment as previously mentioned). We also considered a subanalysis consisting of placebo group who are vitamin D insufficient (<75 nmol/l) both at the start and the end of the study versus. active vitamin D group who were insufficient at the start and became sufficient at the end of the study. These “modified” placebo and active vitamin D groups consisted of 33 and 30 patients, respectively. When we repeat the analysis done in Table 2, the rank sum *P*-values (comparing active vitamin D with placebo) are all nonsignificant, with *P* > .30 for all 10 parameters.

### 3.4. Adverse Events

A total of 72 adverse events were reported in the study over 3 months, 38 in the active vitamin D group and 34 in the placebo group, *P* = .99. There was no significant difference in the adverse events between the study groups. There were 3 serious adverse events, one in the active vitamin D group and two in the placebo group. None of the serious adverse events were considered to be related to the study medication. There were no episodes of nephrolithiasis or hypercalcemia.

## 4. Discussion

The present study did not show an effect of vitamin D3 supplementation on the circulating levels of certain cytokines. We predicted that the levels of IL-2, 6, IFN-*γ*, TNF-*α*, and GM-CSF will decrease under the influence of vitamin D, while the levels of IL-4, 5, 10, and 13 were expected to rise [23–25]. We did not find that cytokine levels or changes in cytokine levels were responsive to vitamin D supplementation or related to serum 25(OH)D levels. However, there is accumulating evidence that vitamin D does significantly alter the immune system [26]. In gene chip experiments, the active form of vitamin D has been shown to downregulate gene expression of T-helper 1 cells, at the same time up regulating gene expression of T-helper 2 cells [24]. Decreased production of INF-gamma and IL-12 by T-helper 1 cells, and induction of regulatory T cells influenced by 1,25(OH)_2_D regulates immune responses to infections [23]. 1,25(OH)_2_D has been shown to induce the expression of IL-10 by CD40/IL-4-activated B-cells by binding of the VDR to the promoter of IL-10 [25].

The role of 1,25(OH)_2_D in innate and adaptive immunity has also been demonstrated in vivo. Following in vivo stimulation of murine dendritic cells by lipopolysaccharide (bacterial-derived endotoxin), they were found to produce 1-alpha-hydroxylase, resulting in production of active form of vitamin D [27]. Hansdottir et al. proposed a mechanism by which vitamin D plays a role in host defense by demonstrating the conversion of inactive vitamin D (25(OH)D) to active 1,25(OH)_2_D by enzyme 1alpha-hydroxylase in respiratory epithelial cells [28]. Impaired production of 1,25(OH)_2_D due to low-circulating 25(OH)D levels results in inhibition of VDR-dependent innate immune response [8]. 

1,25(OH)_2_D inhibits the production of IL-12, IFN-*γ* and promotes production of IL-4 following endotoxin stimulation in rats [24]. Human B-cells experiments demonstrated the ability of 1,25(OH)_2_D to induce the expression of chemokine (C-C motif) receptor 10 (CCR10), which plays an important role in the mucosal immune system [29].

In patients with severe congenital neutropenia, 1,25(OH)_2_D induced the expression of the human cathelicidin antimicrobial peptide 18 precursor protein in myeloid precursors [30]. The process of activation of vitamin D in human tracheobronchial cells causes upregulation of cathelicidin antimicrobial peptide gene and the TLR coreceptor CD14, contributing to innate immunity in the lungs [28].

Data on vitamin D regulating serum cytokines in placebo-controlled clinical trials is very limited. In a 6-month double-blind, placebo-controlled, randomized trial in vitamin D-insufficient patients with MS, 25 *μ*g (1000 IU) vitamin D significantly increased serum levels of TGF-beta 1, while having no effect on TNF-*α*, IFN-*γ*, and IL-13 [31]. Another randomized, placebo-controlled clinical trial in 123 CHF patients demonstrated that 50 *μ*g (2000 IU) of vitamin D, administered for 9 months, increased median serum levels of anti-inflammatory IL-10 by 43%, and suppressed the levels of proinflammatory TNF-*α* [16]. The median baseline 25(OH)D levels in that trial were significantly lower compared to our study: 35.87 nmol/l in vitamin D group and 38.09 nmol/l in the placebo group. Seasonal differences in cytokine levels were observed by Stewart et al. among healthy controls, who noted lower winter levels of IL-4, IL-10 and TNF-*α*, and a winter excess of IFN-*γ* [32]. These findings further support the hypothesis that seasonal differences in vitamin D influence the levels of cytokines and may help explain our finding of declining serum cytokines levels during winter months in the active vitamin D, as well as the control group.

The strengths of this study include the high compliance rate of medication intake (94%) and low-dropout rate. The mean 25(OH)D level of the subjects at baseline (63.7 ± 28.7 nmol/L)  was in accord with the mean 25(OH)D level in NHANES III (64.8 nmol/L) measured during the winter [33]. Our laboratory participates in the international Vitamin D External Quality Assessment Program which ensures the analytical reliability of 25(OH)D assays. Vitamin D content in the tablets was verified by an independent laboratory.

We recognize that this study has several limitations. 50 *μ*g/day may not be enough to stimulate innate immunity, and/or the length of the study may not have been sufficient to observe an effect. Another reason why we may not have observed a benefit is that the mean 25(OH)D level at the beginning of the study was not that low but it was not different from the average level seen in NHANES III. Vitamin D repletion in subjects who were deficient at baseline may have resulted in meaningful changes in cytokine levels however, our study was not designed to address this question. 73% of patients in the vitamin D group achieved 25(OH)D levels greater than 75 nmol/L but the difference between baseline and end-of-study levels may not be enough to confer a benefit. 

Although there is an increasing amount of evidence from animal experiments and human case-control studies that demonstrate the role of vitamin D in host defense, additional randomized clinical trials are needed [34].

## Figures and Tables

**Table 1:** Baseline characteristics of study participants. There were no significant differences between the study groups at baseline.

Characteristic	Active (*n* = 78)	Placebo (*n* = 70)
*Age, y	59.3 ± 13.0	58.1 ± 13.4
BMI, kg/m^2^	26.1 ± 4.5	26.6 ± 4.1
**Male	17 (21.8%)	13 (18.6%)
Female	61 (78.2%)	57 (81.4%)
Race		
Caucasian	70 (89.7%)	61 (87.1%)
African American	3 (3.8%)	3 (4.3%)
Asian	2 (2.6%)	6 (8.6%)
Other	3 (3.8%)	0 (0.0%)
*25-OHD, nmol/L	64.3 ± 25.4	63.0 ± 25.8
PTH, pg/mL	29.2 ± 13.6	28.4 ± 12.6
History of tobacco use	26 (33.3%)	28 (40%)
History of asthma	6 (7.7%)	2 (2.9%)
History of COPD	3 (3.8%)	2 (2.9%)
Received flu vaccine	44 (56.4%)	45 (64.3%)
Dietary calcium intake (mg/d)	762.8 ± 375.7	854.6 ± 518.6
Dietary vitamin D intake (IU/d)	147.3 ± 182.3	168 ± 146.5

*Values are expressed as mean ± SD.

**Values are expressed as *n* (%).

Epidemiology and Infection, see [19] © 2009 Cambridge University Press.

**Table 2:** Laboratory values.

	Active (*n* = 63)			Placebo (*n* = 57)			Rank-sum *P*-value
Cytokine	Median baseline value (in pg/ml)	Median percent change (12 weeks)	*P*-value (signed-rank test)	Median baseline value (in pg/ml)	Median percent change (12 weeks)	*P*-value (signed-rank test)	
GM-CSF	5.91	−63.0	<.0001	4.78	−53.2	.0007	.53
IFN-*γ*	130.8	−38.9	<.0001	115.2	−34.4	.0011	.41
IL-2	2.13	−17.6	.70	2.15	−23.8	.52	.96
IL-4	6.10	−50.8	.001	8.26	−39.4	.006	.47
IL-5	4.88	−8.32	.49	4.53	−30.0	.66	.50
IL-6	2.19	−43.2	.37	2.75	−52.9	.0963	.61
IL-8	16.3	−48.4	<.0001	24.1	−33.0	.0210	.21
IL-10	6.82	−70.4	<.0001	5.12	−49.6	.0401	.02
IL-13	0.604	−59.4	.0104	0.500	−14.4	.22	.21
TNF-*α*	5.64	−12.0	.21	7.60	−21.8	.0542	.64

**Table 3:** Median Difference of Percent Changes (Active vitamin D-Placebo).

Cytokine	Difference of MedianPercent Change (Active vitamin D-Placebo)	95% CI of Difference of Medians
GM-CSF	−3.86%	(−19.9%, 9.69%)
IFN-*γ*	−5.24%	(−18.6%, 8.47%)
IL-2	0.21%	(−31.4%, 26.9%)
IL-4	−5.59%	(−27.2%, 9.10%)
IL-5	8.62%	(−17.6%, 41.0%)
IL-6	4.14%	(−12.5%, 24.4%)
IL-8	−9.43%	(−27.2%, 5.15%)
IL-10	−17.0%	(−38.6%, − 1.15%)
IL-13	−9.08%	(−48.7%, 4.71%)
TNF-*α*	−5.67%	(−38.1%, 23.9%)
